# Prognostic factors in solitary plasmacytoma of the bone: a multicenter Rare Cancer Network study

**DOI:** 10.1186/1471-2407-6-118

**Published:** 2006-05-05

**Authors:** David Knobel, Abderrahim Zouhair, Richard W Tsang, Philip Poortmans, Yazid Belkacémi, Michel Bolla, Fazilet Dinçbas Oner, Christine Landmann, Bernard Castelain, Mahmut Ozsahin

**Affiliations:** 1 Department of Radiation Oncology, Centre Hospitalier Universitaire Vaudois, Lausanne, Switzerland; 2 Department of Radiation Oncology, Princess Margaret Hospital, Toronto, Canada; 3 Department of Radiation Oncology, Dr. Bernard Verbeeten Institute, Tilburg, The Netherlands; 4 Department of Radiation Oncology, Hôpital Tenon, Paris, France; 5 Department of Radiation Oncology, Centre Hospitalier Universitaire, Grenoble, France; 6 Department of Radiation Oncology, Cerrahpasa School of Medicine, Istanbul University, Turkey; 7 Kantonsspital, Basel, Switzerland; 8 Department of Radiation Oncology, Centre Oscar-Lambret, Lille, France

## Abstract

**Background:**

Solitary plasmacytoma (SP) of the bone is a rare plasma-cell neoplasm. There are no conclusive data in the literature on the optimal radiation therapy (RT) dose in SP. Therefore, in this large retrospective study, we wanted to assess the outcome, prognostic factors, and the optimal RT dose in patients with SP.

**Methods:**

Data from 206 patients with bone SP without evidence of multiple myeloma (MM) were collected. Histopathological diagnosis was obtained for all patients. The majority (n = 169) of the patients received RT alone; 32 chemotherapy and RT, and 5 surgery. Median follow-up was 54 months (7–245).

**Results:**

Five-year overall survival, disease-free survival (DFS), and local control was 70%, 46%, and 88%; respectively. Median time to MM development was 21 months (2–135) with a 5-year probability of 51%. In multivariate analyses, favorable factors were younger age and tumor size < 5 cm for survival; younger age for DFS; anatomic localization (vertebra vs. other) for local control. Older age was the only predictor for MM. There was no dose-response relationship for doses 30 Gy or higher, even for larger tumors.

**Conclusion:**

Younger patients, especially those with vertebral localization have the best outcome when treated with moderate-dose RT. Progression to MM remains the main problem. Further investigation should focus on adjuvant chemotherapy and/or novel therapeutic agents.

## Background

Solitary plasmacytoma (SP) is a rare plasma-cell neoplasm [1]. There are two separate entities, dependent on the location of the lesion originating in either bone or extramedullary soft tissue [2]. It is defined as a proliferation of monoclonal plasma cells without evidence of significant bone-marrow plasma-cell infiltration [1]. Bone SP is characterized by a unique lesion involving any part of the squeleton, most commonly in the spine.

Recommendations for the choice of treatment modalities in this radiosensitive disease [3] are based mainly on data from retrospective studies often considering relatively small numbers of patients, with a limited ability to make any robust conclusions [4,5], and only one prospective study including 53 patients [6].

Definitive radiation therapy (RT) is the treatment of choice for solitary plasmacytoma giving adequate local control. However, with respect to long-term outcome, it is known that bone plasmacytoma progresses more frequently to multiple myeloma (MM) than soft-tissue plasmacytoma [7-9].

There are no conclusive data in the literature on the optimal RT dose in SP. Many authors reported a wide range of doses varying from 30–66 Gy [10-12]. Most centers use 50 Gy or more to treat SP, without any strong evidence whether doses higher than 30 Gy are needed.

We report herein the results of a retrospective multicenter study of a large cohort of patients with bone SP from European and North American centers, assessing treatment approaches, radiation dose-response effects and different prognostic factors for survival, myeloma progression, and patterns of local failure.

## Methods

Between 1977 and 2001, 206 adult patients consecutively treated for a bone SP without evidence of MM were collected in a retrospective multicenter study of the *Rare Cancer Network *(RCN). The review board of the RCN granted ethical approval for this study, and individual centers obtained individual ethical approval according to their local regulations for retrospective studies. The study included all patients with a new diagnosis of bone SP and considered candidates for curative RT. According to the new International Myeloma Working Group (IMWG) criteria [13], a patient can be considered to have a bone SP if there is no M-protein in serum and/or urine (a small M-component may sometimes be present); only a single area of bone destruction related to clonal plasma cells; no bone marrow infiltration; normal skeletal survey including spine and pelvic MRI; and no end-organ damage other than solitary bone lesion. Our study, which was designed and conducted before the publication of the IMWG guidelines, only the following criteria were used: a patient was considered to have a bone SP if there is a biopsy-proven solitary bone lesion; <10% plasma cells in the bone-marrow biopsy; no additional pathological lesions in the skeletal diagnostic work-up; no anemia; and normal blood chemistry. The median age was 60 years (range: 18–87), and the male-to-female ratio was 1.82. Pathological diagnosis was obtained in all patients, by biopsy in 137, partial resection in 66, and complete resection in 9 patients. Spinal column was the most frequent tumor site (n = 102). Initial diagnostic work-up was done according to standard recommendations current at the time of diagnosis. Magnetic-resonance imaging (MRI) was used only in 76 patients (37%). The largest diameter of the lesion was recorded in 104 patients (50%), and its median value was 5 cm (range: 1–18 cm). It was 5 cm or more in 59 patients (29%), and less than 5 cm in 45 (22%). Serum immunoglobulin levels, a factor which indicates the possibility of occult disseminated disease, were reported in 120 patients (median 6.5 g/l; range: 0–82); however, myeloma (M) protein levels were not assessed following RT [11]. In this series, serum immunoglobulin level more than 20 g/l was recorded only in 18 patients. Serum electrophoresis and immunofixation were performed in 131 patients. Beta-2 microglobulin data were recorded only for 60 patients. Human immunodeficiency-virus testing was done in only 5 patients, and all were negative. Table 1 summarizes the characteristics of all patients. Median follow-up was 54 months (range: 7–245).

### Treatment

All but 5 patients received RT. Surgical treatment was implemented in 69 (33%) patients (partial resection in 66, and complete excision in 3). Chemotherapy was given in 32 (16%) patients. The planning RT volume included the radiologically visible gross tumor volume with a sufficient margin. For vertebral SP, the planning RT volume included one vertebra above and below the involved vertebra. No attempt was made to cover regional lymph nodes. Median dose was 40 Gy (range: 20–64); median number of fractions was 20 (range: 4–33) using a median dose of 2 Gy per fraction (range: 1.25–5). To facilitate the comparison of the different fractionation schedules used by different centers, we computed the 2-Gy per fraction biologically effective dose (BED) according to the linear-quadratic model: BED = nd(1+ [d÷α/β]); where α/β = 10 for plasmacytoma; n = number of fractions; and d = dose per fraction [14]. Median BED was 40.00 Gy (range: 18.75–66.00).

Six cycles (range: 1–12) of chemotherapy consisting of melphalan and prednisone (n = 23; 72%), vincristine, doxorubicin and dexamethasone (VAD) (n = 7; 22%), and other combinations (n = 2; 6%) were administered in 32 (16%) patients.

### Statistical methods

Overall survival (OS), disease-free survival (DFS), local control, and time to MM progression were estimated using the product-limit method [15]. Time to any event was measured from the date of histological diagnosis. The events were death (all causes) for OS, and death (all causes) or relapse and/or progression to MM for DFS. For the local control rate, the event consisted of local failure, censoring patients without local relapse. For progression to MM, all patients without MM progression were censored. Confidence intervals (CI) were calculated from standard errors. Differences between groups were assessed using the log rank test [16]. Multivariate analyses were done using the Cox stepwise-regression analysis to determine the independent contribution of each prognostic factor [17].

## Results

### Local control

**Figure 1:**
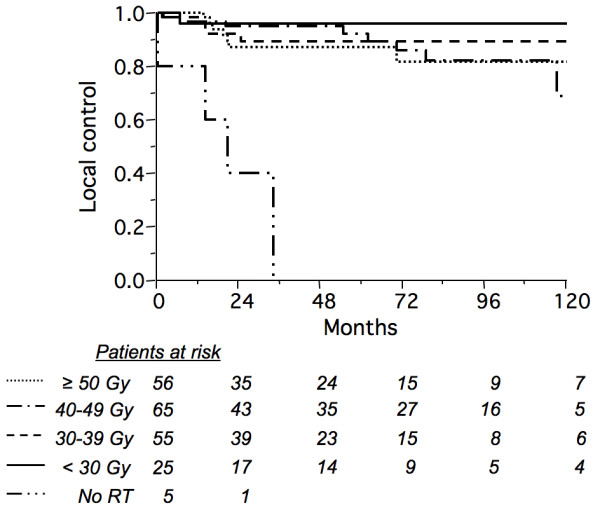
Actuarial local control rate according to biologically equivalent dose at 2 Gy per fraction (no radiation therapy: solid/double-dot line; <30 Gy: solid line; 30–39 Gy: dashed line; 40–49 Gy: solid/single-dot line; = 50 Gy: dotted line) in 206 patients with solitary bone plasmacytoma (p < 0.0001 but not significant when excluding non irradiated patients).

Median time to local relapse was 20 months (range: 2–148), and was observed in 13% of the patients (n = 27). No regional lymph node relapse was observed. The 5- and 10-year probability of local control was 88% (95% CI: 83–93) and 79% (95% CI: 69–89), respectively. Ten-year local control according to the RT dose and to other candidate factors is shown in Tables 2 and 3. When considering patients with tumor diameters of 5 cm or larger (n = 59), likewise there was no clear benefit from higher doses. However, patients with vertebral lesions seemed to have a better outcome in terms of local control (Tables 3 and 4). There were 4 (80%) local relapses out of 5 patients who were treated with surgery with or without chemotherapy compared to 21 (14%) out of 148 who received RT alone, and 2 (7%) out of 30 who received RT and chemotherapy. Surgery (RT *vs*. partial or complete resection and RT) did not influence the 10-year probability of local control (80% [95% CI: 71–89] *vs*. 74% [95% CI: 47–100], p = 0.35). After adjusting RT dose to 2 Gy per fraction biologically equivalent dose, no dose-response relationship was observed for doses higher than 30 Gy (15 x 2 Gy; Fig. 1, Table 2). Among 201 irradiated patients, there were 2 (7%) local failures out of 29 patients treated with = 30 Gy *vs*. 20 (12%) out of 168 treated with > 30 Gy.

### Progression to multiple myeloma

**Figure 2:**
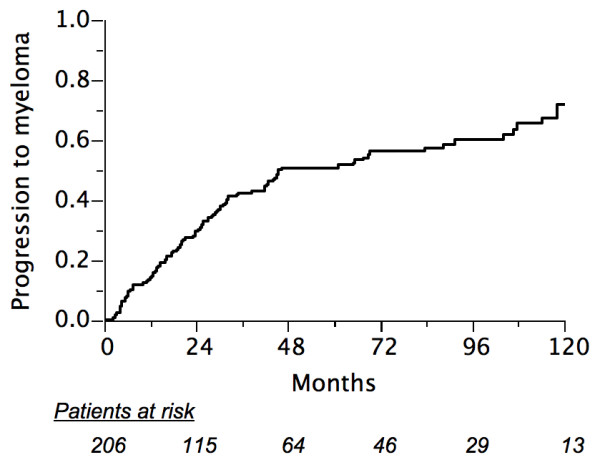
Actuarial probability of progression to multiple myeloma in 206 patients.

Median time to development of MM was 21 months (range: 2–135). One hundred and forth (104) patients developed MM with a 5- and 10-year projected probability of 51% (95% CI: 43–59) and 72% (95% CI: 62–82), respectively (Fig. 2). Among the 120 patients where baseline immunoglobulin levels were recorded (median: 6.5 g/l; range: 0–82), 10 of 18 patients whose levels were above 20 g/l developed MM progression compared to 53 of 102 whose immunoglobulin levels were 20 g/l or less (chi-square: 0.079; p = 0.78). Moreover, in a logistic regression analysis assessing the influence of baseline serum immunoglobulin level on the development of MM, no significant correlation was found (R-square: 0.002; p = 0.60). Ten-year probability of progression to MM according to the type of SP and to other factors is shown in Table 3. The only unfavorable factor for myeloma development was older age (> 60 years) in univariate analyses. The 10-year probability of progression to MM was not influenced by anatomical location (79% for vertebra *vs*. 67% other; p = 0.73). MRI examination during staging work-up was not significantly associated with the 10-year probability of progression to MM (71% in both groups, p = 0.22).

### Survival

At the time of analysis, 135 patients were alive (50 with disease), and 71 had died (54 died of disease including 2 with unknown causes, 16 related to intercurrent disease, and 1 from cardiopulmonary disease probably related to mediastinal RT).

In all patients, the 5- and 10-year probability of OS were 70% (95% CI: 63–77) and 50% (95% CI: 40–60), respectively; and for 5- and 10-year DFS, 46% (95% CI: 38–54) and 25% (95% CI: 16–34), respectively.

In univariate analyses, statistically significant best factors influencing the OS were younger age (60 years or younger), and tumor size (< 5 cm). Ten-year probabilities of OS and DFS according to different prognostic factors are shown in Table 3.

### Multivariate analyses

Multivariate analyses revealed that the best independent factors predicting the outcome were: younger age (RR = 0.59; p < 0.00001) and tumors less than 5 cm (RR = 0.56; p = 0.0007) for OS; younger age (RR = 0.79; p = 0.02) for DFS; and vertebra localization (RR = 0.63; p = 0.04) for local control. Older age (RR = 0.78; p = 0.01) was the only independent predictor for MM development.

### Toxicity

Early side effects according to CTC v2.0 depended on the irradiated anatomical regions [18]. Grade 1 toxicity was observed in 46 patients (22%), grade 2 in 14 (7%), and grade 3 in 4 (2%).

According to the RTOG/EORTC late effect scoring system [19], grade 1 toxicity was observed in one patient (asymptomatic bone necrosis), no grade 2 toxicity, and grade 3 toxicity in 2 (femoral head necrosis in one, and xerostomia in the other). Grade 4 pulmonary toxicity was observed in one patient who died from cardiopulmonary disease probably related to mediastinal RT toxicity.

## Discussion

We recently reported our experience on a series of 258 patients including bone and extramedullary SP [20]. In the present study, we wanted to specifically assess different treatment approaches, radiation dose-response effects; and different prognostic factors in patients with bone plasmacytoma. Several factors are reported to influence the outcome of SP in the literature [2,8,10,12,20-24].

Despite good local control with RT, patients with bone SP progresses to MM more frequently than those with extramedullary SP [7,10]. Many questions remain unanswered about prognostic factors influencing the outcome and radiation dose-response, probably due to the limited number of patients included in reported series [9,12,24]. This may be related to the lack of routine use of MRI to exclude occult lesions at initial diagnosis [25,26]. MRI was done in only 33% of our patients (Table 1). Staging using MRI to exclude multiple lesions has contributed in recent years to more accurate diagnosis of SP, since only the patients with truly solitary involvement are eligible for curative RT [25,26]. This may also help radiation oncologists to define target radiation volumes with higher precision [27]. Unfortunately, in this study, whether MRI examination was done or not did not result in an improved ability to predict progression to MM. However, it was not clear how often MRI was done to define the local extent of disease, rather than for the detection of occult disease in other bony sites such as the spine and pelvis. Nevertheless, in our patients who had an MRI examination, no occult asymptomatic MM lesions were detected.

There is limited information in the literature regarding the outcome of elderly patients with myeloma. Some series indicate that advanced age is clearly associated with poor survival whereas others do not [28]. In our series older age (>60 years) was found to be a bad prognostic factor, either in univariate or multivariate analyses, in terms of OS, DFS, or progression to MM. This difference may be related to several reasons. In terms of survival, older patients who develop MM are possibly treated with "less aggressive" treatments, or not included in prospective trials compared to younger patients [29]. However, it is difficult to find an explanation why our patients older than 60 years develop MM more frequently than the younger ones.

Patients in this study seemed to develop MM in two peaks. A first peak during the first three years post treatment is probably due to undetected asymptomatic gross disease becoming more obvious; a second peak corresponding to a progression of previously occult disease is observed after 6–9 years (Fig. 2). A number of patients showed very late myeloma progression, even after 10 years. Of note, while vertebral disease has been reported to be a poor prognostic factor compared to other bony localizations [7], in our series 50% of bone SP were located in the axial skeleton but no difference in terms of progression to MM was observed. However, in multivariate analysis, patients in our series with vertebral lesions had a better local control compared to other anatomical locations (Table 4).

Progression to MM has been reported to be less frequent in younger patients, whose survival is also better [10,24,30,31]. Our study confirmed these reports, with progression to MM seen more frequently in older patients, and improved survival in younger patients (Fig. 3, Table 3). However, local failure did not depend on age.

Baseline immunoglobulin (M protein) levels are reported to be a predictive factor of occult disseminated disease, and almost all experts agree that an M protein level of 20 g/l is crucial for the definition of SP [11,13]. In our series, only 18 patients had an M protein level above 20 g/l, and no difference in terms of MM development was observed between patients with M protein levels above and below 20 g/l. Furthermore, patients showing persistent M protein levels for more than one year after RT are prone to progress to MM [30,32]. This factor could not be assessed in our series because we did not have adequate data on M protein level responses to treatment, and the correlation of progression to MM with M protein persistence.

Tumor size is reported to be an important prognostic factor in terms of local control. Tsang et al [10] reported a better outcome for lesions less than 5 cm, while other studies did not find tumor size to be a factor [32]. In our series, local control was higher for plasmacytomas measuring less than 5 cm (91%) than for those 5 cm or higher (73%). It is important to note that size was not reported for 112 (43%) of the patients in our study (Table 3).

In a large review of over 400 publications from 1905 to 1997, Alexiou et al [33] reported evidence that surgery alone gave the best results for extramedullary bone plasmacytoma when clear surgical margins were obtained. In the present series, 94 patients were treated by surgery with (n = 86) or without RT (n = 8). Nine (3%) had a complete resection with negative margins; of these, only one received adjuvant RT, and seven relapsed. This finding argues against proposing surgery alone, even in cases where complete resection is achieved.

While radiation therapy is the treatment of choice for solitary plasmacytoma, the evidence of its efficacy is solely based on small retrospective studies [34]. There is no clear RT dose-response relationship in the literature. The analysis performed by Mendenhall et al [22] suggested a minimum dose of 40 Gy for optimal local control. Despite little evidence for a dose-response relationship above 30–40 Gy [8,21,24], some authors propose doses between 40 and 50 Gy for small lesions, and higher doses for larger tumors [23,26,34]. While Tsang et al [10] reported that there was no convincing dose-response relationship above 35 Gy for small lesions, they concluded that local control was related to the size of the lesion and suggested giving higher doses or combined modality treatment for bulky tumors. This has also been proposed by other groups [8].

Our data showed no correlation between local failure and radiation dose, even for large tumors (Fig. 1, Table 2). Due to its retrospective nature, these results should be interpreted with caution. However, it included a large number of patients treated with a wide spectrum of RT doses, with the finding that 30 Gy in 2-Gy fractions, or a biologically equivalent regimen was sufficient. It appears from this study that administering a higher radiation dose may be unable to overcome the negative prognostic impact of a bulky tumor on local control, in comparison with smaller tumors (Table 2). In addition to size, it is possible that other poorly documented factors (e.g. local invasiveness, proliferation rate, or morphologic grade) also impact on the risk of local relapse, and are confounding factors in a dose-response analysis.

Chemotherapy, which is not considered as a standard of care in patients with SP, has been proposed by some investigators in the initial management of bone plasmacytoma. Only one prospective study reported a benefit with combined chemotherapy and RT compared to RT alone [6]. The chemotherapy used consisted of melphalan-prednisone given every six weeks for three years. Although a limited number of patients was included (n = 53), this study concluded that combined radiochemotherapy seems to increase remission and survival duration.

Targeting the mechanisms that control angiogenesis, which has an integral role in the pathophysiology of hematologic malignancies, could be an innovative therapeutic approach in the treatment of SP. Kumar et al [35] reported high-grade angiogenesis in 64% of tumors in their series of 25 bone SP patients, and found that angiogenesis is highly correlated with progression to MM. Therefore, antiangiogenic compounds such as thalidomide, vascular-endothelial growth-factor, or proteasome inhibitors may be promising in this disease.

## Conclusion

Overall, the results obtained in this study, to our knowledge the largest series in the literature, are in accordance with other published series [4,5,8,9,12,21,23,24]. Although high rates of local control were obtained, survival was disappointing because of progression to MM. Only a large prospective study conducted by collaborative groups could answer the unresolved questions in this relatively rare disease. Following a complete staging work-up, which may include very sensitive imaging tools such as positron-emission tomography [36,37], current evidence supports the use of involved-field moderate-dose radiation therapy. Future investigation in phase II or III prospective trials should focus on the use of adjuvant chemotherapy and/or novel therapeutic agents.

## Abbreviations

SP: solitary plasmacytoma

MM: multiple myeloma

RT: radiation therapy

MRI: magnetic-resonance imaging

BED: biologically effective dose

OS: overall survival

DFS: disease-free survival

CI: confidence intervals

CTC: common toxicity criteria

RTOG: Radiation Therapy Oncology Group

EORTC: European Organisation for Research and Treatment of Cancer

## Competing interests

The author(s) declare that they have no competing interests.

## Authors' contributions

DK, AZ, RWT, PP, YB, MB, FOD, CL, BC, and MO included patients in the study. DK, AZ, and MO realized the conception and design of the study. MO did the statistical analysis and interpretation of the data. MO and AZ drafted the manuscript, and RWT revised it critically. All the authors have read, revised, and approved the manuscript.

## Pre-publication history

The pre-publication history for this paper can be accessed here:



## Figures and Tables

**Table 1:** Characteristics of 206 patients with bone plasmacytoma

	Number of patients	Percentage
*Staging Work-up*		
Bone-marrow assessment	206	100
Standard X-rays	167	82
Computer tomography	135	67
Serum immunoglobulins	120	58
Immunosubtraction	131	64
MRI	76	37
Bone scintigraphy	60	29
*Site of lesion*		
Vertebrae	102	50
Pelvis	26	12
Ribs	18	9
Upper extremities	16	8
Maxillary bone	13	6
Skull	11	5
Lower extremities	10	5
Sternum	10	5
*Surgical intervention*		
Biopsy	137	67
Partial resection	66	32
Complete resection	3	1
*Treatment*		
RT alone	169	82
CT+RT	32	16
Surgery alone (+CT in one)	5	2

MRI: magnetic resonance imaging, RT: radiation therapy, CT: chemotherapy.

**Table 2:** Distribution of local relapses according to biologically effective radiation dose*

All patients (n = 206)			Tumors ≥ 5 cm (n = 59)	
Radiation dose (Gy)	Number of patients	Local relapse (%)	Number of patients	Local relapse (%)

No RT**	5	4 (80%)	1	1 (100%)
<30	25	2 (8%)	2	0
≥ 30 – <40	55	5 (9%)	17	3 (18%)
≥ 40 – <50	65	8 (12%)	15	3 (20%)
≥ 50	56	8 (13%)	24	4 (17%)

RT: radiation therapy

*biologically effective dose (BED) according to the linear-quadratic model.^13^

BED = nd(1+ [d÷α/β]) where n = number of fractions, d = dose per fraction, and α/β = 10 for plasmacytoma.

**All patients treated with surgery alone (with chemotherapy in one).

**Table 3:** Univariate analyses (logrank test)

	n	10-yr. OS (%)	%95 CI (%)	p-value	10-yr. DFS (%)	%95 CI (%)	p-value	10-yr. LC (%)	%95 CI (%)	p-value	10-yr. MM (%)	%95 CI (%)	p-value
													
*All patients*	206	52	43–61		25	15–35		79	69–89		72	62–82	
													
**Age (year)**													
≤ 60	103	63	50–76	<0.0001	29	16–42	0.02	79	64–94	0.46	67	53–81	0.007
>60	03	34	19–49		22	9–35		79	67–91		76	62–90	
													
*Gender*													
Female	73	52	36–68	0.98	38	23–53	0.79	88	79–97	0.60	58	43–73	0.77
Male	133	49	37–61		23	13–33		75	62–88		74	63–85	
													
*Site of plasmacytoma*													
Vertebra	102	52	38–66	0.99	19	4–34	0.89	89	79–98	0.07	79	64–94	0.39
Other	104	48	35–61		28	16–40		78	68–88		66	53–79	
													
*Largest tumor dimension (cm)*													
<5	45	67	36–98	0.001	24	7–41	0.73	87	75–99	0.47	79	58–100	0.31
≥ 5	59	57	42–72		34	19–49		78	65–91		58	42–74	
NA	102	33	19–47		16	0–33		73	50–96		80	64–96	
													
*IgM subtype*													
Kappa	59	49	19–47	0.57	0	-	0.57	60	24–94	0.49	91	75–100	0.36
Lambda 100	31	61	40–81		35	16–54		87	69–100		65	46–84	
NA	116	49	37–61		22	20–44		84	75–93		64	52–76	
													
*Type of treatment*													
RT alone	169	51	40–62	0.49	27	16–38	0.92	78	66–90	0.27	71	60–82	0.93
CT + RT 100	32	43	20–66		15	0–39		93	84-		77	54–100	
													
*2 Gy/fr equivalent RT dose (Gy)*^a^													
≥ 50	56	47	28–66	0.50	26	10–42	0.06	82	68–96	<0.0001^b^	71	55–87	0.25
≥ 40 and <50	65	55	34–76		25	6–44		67	40–94		78	59–97	
≥ 30 and <40	55	46	28–64		32	16–48		90	81–99		63	47–79	
<30	25	37	13–61		17	0–43		96	88–100		81	53–100	
No RT^c^	5	0	-		0	-		0	-		33	0–86	

OS: overall survival; CI: confidence interval; DFS: disease-free survival; LC: local control; MM: progression to multiple myeloma; NA: not available; CT: chemotherapy; RT: radiation therapy ^a^biologically effective dose (BED) according to the linear-quadratic model.^13 ^BED = nd(1+ [d÷α/β]) where n = number of fractions, d = dose per fraction, and α/β = 10 for plasmacytoma. ^b^not significant when excluding patients not receiving radiation therapy. ^c^All patients treated with surgery alone (with chemotherapy in one).

**Table 4:** Multivariate analysis* (Cox model) in 201 irradiated patients

Covariable	OS		DFS		LC		MM		
	RR	*p-value*	RR	*p-value*	RR	*p-value*	RR	*p-value*	comment
Age (years)	0.59	<0.00001	0.79	0.02	-	-	0.78	0.01	≤ 60 years better
									
Localization (vertebra *vs.*other)	-	-	-	-	0.63	0.04	-	-	vertebra better
									
Tumor size (cm) (<5 *vs.* ≥5)	0.56	0.0007	-	-	-	-	-	-	<5 cm better

*including age, gender, localization, tumor size, IgM subtype, type of treatment, and biologically equivalent radiation dose.

OS: overall survival; DFS: disease-free survival; LC: local control; MM: progression to multiple myeloma
